# Non-invasive Imaging in Patients With Chronic Total Occlusions of the Coronary Arteries—What Does the Interventionalist Need for Success?

**DOI:** 10.3389/fcvm.2021.713625

**Published:** 2021-08-30

**Authors:** Johannes Kersten, Nina Eberhardt, Vikas Prasad, Mirjam Keßler, Sinisa Markovic, Johannes Mörike, Nicoleta Nita, Tilman Stephan, Marijana Tadic, Temsgen Tesfay, Wolfgang Rottbauer, Dominik Buckert

**Affiliations:** ^1^Department for Internal Medicine II, University of Ulm, Ulm, Germany; ^2^Department for Nuclear Medicine, University of Ulm, Ulm, Germany

**Keywords:** chronic total occlusion, revascularization, non-invasive imaging, hibernation, ischemia, viability

## Abstract

Chronic total occlusion (CTO) of coronary arteries is a common finding in patients with known or suspected coronary artery disease (CAD). Although tremendous advances have been made in the interventional treatment of CTOs over the past decade, correct patient selection remains an important parameter for achieving optimal results. Non-invasive imaging can make a valuable contribution. Ischemia and viability, two major factors in this regard, can be displayed using echocardiography, single-photon emission tomography, positron emission tomography, computed tomography, and cardiac magnetic resonance imaging. Each has its own strengths and weaknesses. Although most have been studied in patients with CAD in general, there is an increasing number of studies with positive preselectional factors for patients with CTOs. The aim of this review is to provide a structured overview of the current state of pre-interventional imaging for CTOs.

## Introduction

Chronic total occlusion (CTO) of coronary arteries is a common finding in coronary angiograms of patients with known or suspected coronary artery disease (CAD). Despite their frequency, CTOs are the most reliable predictor of an incomplete revascularization. This is the result of two major factors: (a) a lack of data from randomized controlled trials regarding a benefit on mortality and (b) the lower success rate accompanied by a higher complication rate of an interventional revascularization. Large registry studies have shown that CTO percutaneous coronary interventions (PCI) can reduce mortality (1–4). The EUROCTO trial, the first randomized controlled trial on CTO, showed a benefit in terms of ischemic symptom burden rather than other hard clinical outcomes (5). In another randomized controlled trial (REVASC Trial), CTO PCI did not show an improvement in analyzed cardiovascular magnetic resonance (CMR) parameters but a reduction in major adverse cardiovascular events (6).

Current guidelines and position papers therefore recommend CTO PCI in the case of ongoing symptoms and viable myocardia in the CTO territory (7–10). However, imaging of ischemia remains controversial. On the one hand, revascularization is recommended for CAD patients with more than 10% myocardial ischemia (7, 8). However, the trial underlying this recommendation focused on patients with CAD in general and not on CTO patients in particular (11). On the other hand, ischemia is always seen in viable CTO-related myocardia regardless of the grade of collaterals, and in most CTOs of major vessels, the degree of myocardial ischemia is above 10% (12, 13). Even though the ISCHEMIA trial found no benefit of an ischemia-driven revascularization approach, its results are not generally applicable to patients with CTOs (14, 15).

Viability is by definition present in segments with preserved systolic function. In contrast, dysfunctional segments are not a priori avital. The term “hibernation” was introduced to explain a potentially dysfunctional myocardium resulting in ischemia. Information is gained from an initial coronary angiogram where well-developed collaterals correlate with less extensive scarring, indicating a more viable myocardium (16, 17). A normal wall motion in CTO-related areas is a common finding, and extensive, transmural scarring is only observed in about 5% of CTO segments (16). Otherwise, the presence or extent of a Q wave or QS complexes in an electrocardiogram (ECG) is no proof of a vitality (18–20).

**Figure 1:**
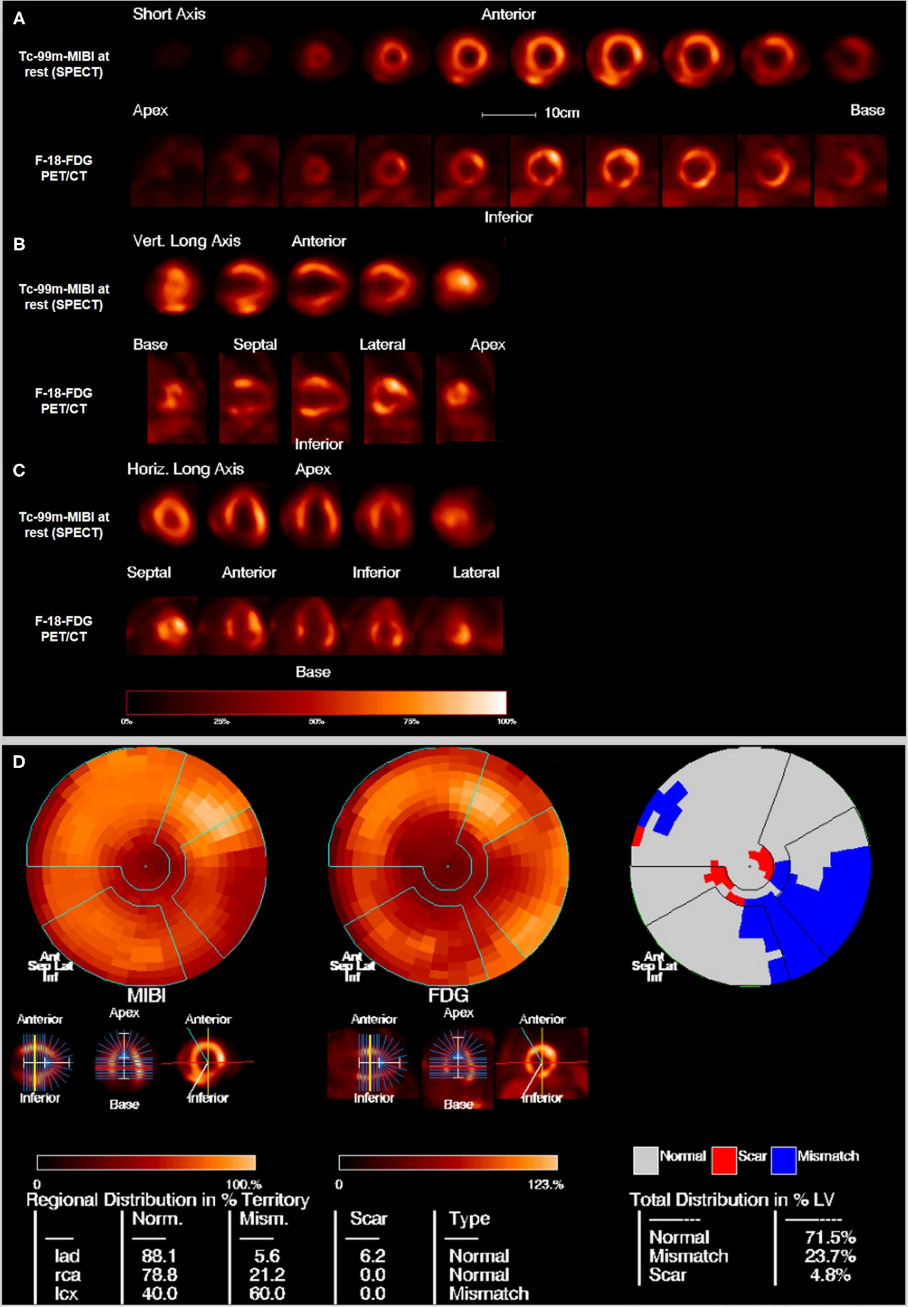
Example of hibernating myocardium: 76 years old male with a perfusion defect on the posterolateral wall in the Tc-99m-MIBI rest scan that shows FDG uptake in the viability F-18-FDG-PET/CT scan resulting in the diagnosis of hibernating myocardium. **(A)** Short axis, **(B)** vertical long axis, and **(C)** horizontal long axis. The upper rows show the perfusion scan at rest with Tc-99m-MIBI (SPECT), the lower rows show the viability scan with F-18-FDG (PET). **(D)** Polar maps and subtraction image to visualize the mismatch between the perfusion defect on SPECT and the viability on FDG-PET/CT. 23.7% of the myocardium in the left ventricle is in hibernating status.

For the detection of a hibernating myocardium, a broad range of non-invasive imaging modalities is available. An overview is presented in Table 1 with an example of their use in Figure 1. The selection mainly depends on local availability and expertise. This review aims to discuss the possibilities and limits of the available non-invasive imaging modalities and future trends. The review focuses on studies aiming to optimize patient selection for CTO reperfusion and its outcomes, although most findings are applicable to CAD patients in general.

**Table 1:** Viability testing using different non-invasive imaging modalities.

	**DSE**	**SPECT**	**FDG-PET**	**CT (experimental)**	**CMR**
Sensitivity	80–84%	83–87%	88–93%	N/A	LGE: 84–95% Dobutamine: 81–84%
Specificity	78–81%	65–69%	58–73%	N/A	LGE: 51–74% Dobutamine: 82–91%
Radiation dose	None	6–24 mSv	~7 mSv	~7 mSv	None
Contrast agent/tracer	Not necessary	Radioactive tracer (^99m^Tc or ^201^Tl)	Radioactive tracer (^18^F, ^82^Rb)	Iodine	LGE: gadolinium Dobutamine: None
Cost	Low	Moderate	High	Moderate	High
Limitations	Very low image quality in some patients	Low resolution and high radiation dose	High cost and high radiation dose	High radiation dose and limited knowledge of viability testing	High cost and long duration
Advantages	Low cost and wide availability	Balanced cost and diagnostic accuracy	High resolution and sensitivity	Positive adjunctive information	Availability of prognostic data in CTO

*Sensitivity, specificity, and radiation doses are adopted from Henzlova et al. (21), Bax et al. (22), Matsuda et al. (23), Case et al. (24), Romero et al. (25), and Schinkel et al. (26)*.

*Viability was defined as functional recovery after percutaneous coronary intervention or coronary artery bypass grafting in CAD patients*.

*DSE, dobutamine stress echocardiography; SPECT, single-photon emission computed tomography; FDG-PET, ^18^F-fluoro-2-deoxy-D-glucose positron emission tomography; CT, computed tomography; CMR, cardiovascular magnetic resonance; LGE, late gadolinium enhancement*.

## Echocardiography

Echocardiography is inexpensive and available nearly everywhere. It is the standard modality for the evaluation of global and regional cardiac function. Besides pre-interventional examinations, the bedside use of echocardiography in the catheter laboratory provides the opportunity to detect complications during interventions, such as pericardial effusions or intramural hematomas.

However, the value and validity of echocardiography depend on the investigator's experience. In CAD patients, an increase in regional contractility under dobutamine stress (“contractile reserve”) can show viability and improvement of regional function after revascularization (27–29). The mostly older studies on dobutamine stress echocardiography (DSE) included small patient samples with a clear underrepresentation of women. The sensitivity of DSE in predicting an improvement of regional function ranges from 74 to 94% (30). In a relatively large trial involving 318 patients, revascularization in segments with viability on DSE was associated with reduced mortality (31). In another trial, viability assessed by DSE was associated with a relative reduction of mortality by 19.5% in patients with a severe left ventricular dysfunction (LVEF <35%) (32). Because of its wide distribution and cost-effectiveness and the accumulated experience in clinical practice, DSE appears to be a good tool for examining patients with CTOs, provided that the image quality is good. Another advantage is the low rate of side effects of dobutamine stress (33). Other forms of myocardial stress are also possible as an alternative to dobutamine, such as dipyridamole and treadmill exercise (34).

In addition to the examination under dobutamine stress, echocardiography at rest can predict viability under certain circumstances. An end-diastolic wall thickness (EDWT) of >0.6 cm in dysfunctional segments is a marker of hibernation. In a trial involving 45 patients undergoing 2D echocardiography, DSE, and rest-redistribution thallium-201 (Tl-201) tomography before revascularization, an EDWT of >0.6 cm had 94% sensitivity and 50% specificity with a ROC curve similar to the maximum Tl-201 uptake, while DSE increased specificity to 88% (33). More recent studies focusing on strain echocardiography have reported promising results (35, 36). However, the interpretation of deformation parameters depends heavily on image quality and the examiner's experience. For wider use, validation in a prospective cohort is warranted.

## Myocardial Perfusion Scintigraphy

Myocardial perfusion scintigraphy (MPS) is a nuclear medicine technique that is used for around 50 years to assess regional left ventricular myocardial perfusion, diastolic and systolic function as well as to differentiate hibernating myocardium from transmural or non-transmural infarct. Three dimension images of myocardium are acquired after injecting radiopharmaceuticals with high first pass extraction by the myocardium using the single emission computed tomography (SPECT) technique. In majority of the centers the images are additionally corrected for attenuation correction using low dose CT images acquired simultaneously with SPECT images. As radioisotopes preferentially Technetium-99m (Tc-99m), specially in Europe and rarely Thallium-201 (Tl-201) are used (21). Tl-201 as a cyclotron product is injected as Thallium-201-Chloride. The Tc-99m is eluted from a generator and is either labeled with Sestamibi (CardioliteTM) or Tetrofosmin (MyoviewTM). Nowadays Tc-99m-labeled tracers are the tracer of choice because of more favorable imaging characteristics because of higher photon energy (140 keV compared to 69–83 keV) with less attenuation by the tissues around the heart, improved image quality and less radiation exposure (6 mSv vs. 17 mSv) compared to Tl-201 because of a shorter half-life (Tc-99m = 6 h, Tl-201 = 72 h). ECG-gated perfusion images allow the simultaneous assessment of perfusion as well as global and regional function of the left ventricle and are therefore an important tool in the diagnosis and management for CAD. To assess perfusion imaging a comparison between images at rest and images after stress is required. To replicate the normal physiological effect of strenuous exercise on perfusion, radiopharmaceuticals are injected after the patients underwent either physical stress (e.g., by a treadmill) or pharmacological stress (adenosine, regadenoson or dobutamine) tests. Thereafter the resting state images are acquired at a later time point. In patients without hemodynamically relevant myocardial ischemia, both stress and rest scans show a homogeneous distribution of the perfusion tracers. In comparison, patients with hemodynamically relevant stenosis of the coronary vessels, decreased segmental or subsegmental uptake of the tracer is seen in stress which however normalizes itself in the resting phase. A scar of the myocardium shows reduced or no uptake in the scar area both during stress and in rest conditions as a fixed defect.

The rarely used Tl-201 is a potassium analog and uses the Na/K ATPase system of viable myocardial cells. Its initial myocardial uptake is proportional to blood flow and it is rapidly cleared from the blood. After that up to 4 h a re-equilibration takes place when Tl-201 concentration levels are lower in the blood. This is also called redistribution and the process is directly proportional to blood flow to the area and viable myocardial cells. If the stress and rest images show matched homogeneous normal tracer uptake there is no sign of ischemia or infarction. If the tracer uptake during stress is abnormal but with normal uptake during rest / redistribution that is a sign for ischemia. Perfusion defects on both stress and rest images usually means there is a scar. To check that area for hibernating myocardium a reinjection of Tl-201 and another image acquisition after 18–24 h can be carried out. If there is Tl-201 uptake, there is hibernating myocardium in this area. If there is a scar no tracer uptake can be detected (37, 38).

The Tc-99m-labeled radiotracers are monovalent cations that enter cells through their lipophilic characteristics. Their uptake is also dependent on blood flow but as well as on electrochemical gradients of the plasma- and mitochondrial-membranes, the cellular pH and intact energy pathways. In the myocytes they are trapped mainly in the mitochondria with minimal washout and no redistribution in the blood. After the intravenous injection these tracers are first cleared by the liver and excreted through the bile. This makes it impossible to assess the myocardial uptake of the inferior wall right after injection and leads to a delay of 15–45 min before the start of the imaging acquisition, especially for Sestamibi. Tetrofosmin allows earlier imaging acquisition because of lower hepatic uptake. Stress and rest images can be acquired in 2-day and 1-day protocols that show no significant differences (21). For the 1-day protocol the stress images should be acquired first. If there is a homogenous tracer uptake the rest images can be avoided. In the 1-day protocols the injection for the second imaging (rest) higher amount of radioactivity is injected to overcome the shine through from the remaining radioactivity after stress. With the Tc-99m-labeled radiotracers perfusion and ischemia as well as viability can be examined but not hibernating myocardium (39). As Tl-201 is associated with higher radiation exposure, hibernating myocardium are best depicted by using a combination of Tc-99m Tetrofosmin or Sestamibi perfusion scan and F-18-FDG-PET.

The accurate interpretation of the myocardial perfusion imaging with SPECT is crucial. Absolute quantification of perfusion is more common with PET than with SPECT. According to the literature the sensitivity and the specificity of gated myocardial SPECT studies to diagnose clinically significant CAD with Tc-99m tracers is 88.3 and 75.8%, respectively (40). Cutoff values for the uptake of the tracers of > 50% of Tc-99m and > 60% of Tl-201 are commonly associated with regional functional recovery in CAD. However, in the detection of a viable hibernating myocardium, specificity is moderate (49–69%) and can lead to an overestimation of potential functional recovery (22). The presence of an ischemic area on a SPECT scan has shown a negative predictive value for cardiac events (41). In a trial involving patients with CTO of the left anterior descending artery, perfusion defects on SPECT scans before a PCI were associated with an improvement of clinical and functional markers, such as the 6-min walk test, left ventricular volumes and ejection fraction (42). The effect was stronger in patients with reversible perfusion defects than in patients with irreversible defects.

Visual interpretation of three vessels CAD is a challenge for nuclear cardiology field. However, with the development and standardization of protocols as well as advances in the scanner types (e.g., digital scanners or the even newer C-shaped heart-specific gamma cameras) there is a remarkable improvement in the quantification of myocardial wall thickening, contraction, and dilatation as well as in measurement of ejection fraction. Left ventricular dilatation in stress in comparison to rest can be quantified as transient ischemic dilatation (TID). A TID value above 1.3 is generally considered to be significant and can be used for assessment of three vessels disease (43).

However, the comparatively low spatial resolution of SPECT images compared to that of other imaging modalities is a potential disadvantage, as it can lead to overdiagnosis of subendocardial infarctions. Nevertheless, this is not a real disadvantage in the examination of patients with CTOs since transmurality is the main question in this case. There are new predefined criteria for differentiating between transmural and non-transmural infarction in SPECT images (44).

Overall, SPECT appears to be a useful examination modality for patients with CTOs. A radiation dose of 6–24 mSv, depending on the protocol and tracer used (lower radiation doses for Tc-99m and higher radiation doses for Tl-201) (21), as well as expertise, should be taken into consideration.

## Positron Emission Tomography

**Figure 2:**
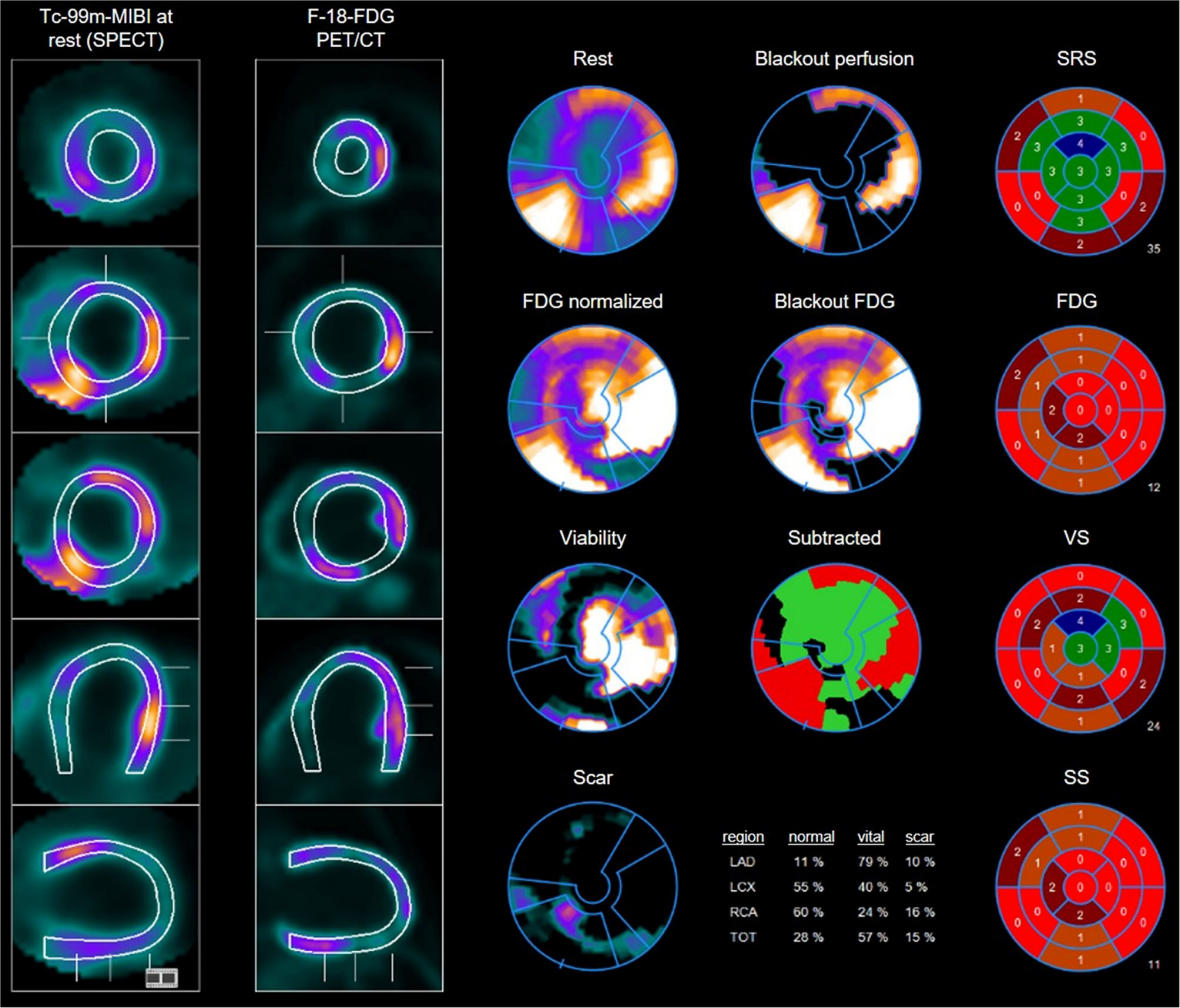
Example of multiple perfusion defect with hibernating myocardium as well as areas of scars: 45 years old male with perfusion defects in the anteroseptal wall as well as on the anterolateral and posterolateral wall in the Tc-99m-MIBI rest scan. FDG uptake in the viability F-18-FDG-PET/CT scan shows some uptake in the areas with perfusion defects resulting in the diagnosis of hibernating myocardium but also some areas with no FDG uptake especially septal and posterior resulting in the diagnosis of scars. Overview and polar maps with SRS, summed rest score; VS, vitality score; SS, scar score.

Positron emission tomography (PET) is another nuclear medicine imaging technique that is in use for around 40 years in some specialized centers with a broader use during the last years. The main advantages of PET over SPECT are higher-quality images because of high-energy emitted photons (511 keV) with improved spatial and temporal resolution and shorter half-lives of the radioisotopes (mostly some minutes) as well as less radiation exposure (45, 46). By the use of F-18-FDG it is also possible to make the myocyte glucose metabolism visible. A combination of MPS and FDG-PET is important in the detection and risk stratification of CAD. Two examples for the detection of hibernating myocardium and the differentiation to scar are seen in Figures 1, 2. Additionally, perfusion PET also allows an improved functional evaluation of CAD because of a higher diagnostic accuracy and the possibility of measurements of myocardial blood flow (MBF) in absolute terms (milliliters per gram per minute). That makes it also possible to detect microvascular disease and balanced ischemia.

For the imaging with PET positron emitting radionuclides like Rubidium-82 (Rb-82), Oxygen-15 (O-15), Nitrogen-13 (N-13), and Flouride-18 (F-18) are used and incorporated into biochemical molecules. Rb-82 is a generator product, the other named radioisotopes are cyclotron products and for O-15 and N-13 with very short half-lives of 2 and 10 min, respectively, making an onsite cyclotron necessary. For perfusion imaging O-15 water, N-13 Ammonia, Rb-82 and F-18 Flurpiridaz are used with stress and rest imaging as in MPS. O-15 water is ideal for quantification of MBF in absolute terms. It can be seen as a one-compartment tracer kinetic model as it has no barrier effect form cellular membranes. Because of its very short half-life and poor contrast images between blood pool and the myocardium its use is limited. N-13 Ammonia is cationic and its first-pass extraction is related to blood flow for low flow rates. It reaches the cytosols via passive diffusion or active transport and is incorporated into the amino acid pool or diffuses back into the blood. Rb-82 is a potassium analog with a very short half-life of 75 s. During first-pass its extraction is not very high and builds a plateau with higher blood flow rates. F-18 Flurpiridaz is a novel PET mitochondrial complex-1 inhibitor in preparation with a half-life of 120 min. It has a high extraction rate with high flow and therefore makes absolute quantification of blood flow possible (47). As for SPECT tracer uptake between stress and rest images are compared to detect ischemia and scars.

For the differentiation of a perfusion defect in both stress and rest between a scar region and ischemic but viable region F-18-Fluorodeoxyglucose (FDG) is used. F-18-FDG competes with glucose for transport as well as phosphorylation by hexokinase. Once it is in the cytosol and phosphorylated it cannot be further processes by glycogen synthesis and is therefore trapped in the cell (45, 48). Its half-life of 110 min makes it easy to handle. To assess myocardial viability of a perfusion defect in stress and rest or in rest only (preferably from PET scans) an additional PET scan with F-18-FDG according to a special protocol can be performed. For non-diabetic and diabetic patients fasting for at least 6 h is required. After measurement of the serum glucose a protocol for glucose loading and insulin is required before F-18-FDG is administered intravenously (glucose <100 mg/dl 25–100 g oral load or dextrose infusion, later insulin is administered according to the glucose level, glucose <250 mg/dl only insulin is administered without an additional glucose load). In a viability study areas with highly reduced or absent perfusion but preserved or even enhanced metabolic activity Perfusion/metabolism mismatch, are hibernating but viable and have a high probability of regaining function after revascularization. Areas that show no perfusion during rest but do have preserved glucose metabolism are viable whereas the absence of glucose metabolism indicates non-viable myocardium. There are three different patterns which can be seen combining myocardial perfusion imaging at rest with F-18-FDG: (1) hibernating myocardium showing reduced perfusion with preserved FDG-uptake, (2) transmural scar without perfusion and without FDG-uptake, and (3) non-transmural scar with partially reduced perfusion of the myocardium with concordant FDG-uptake.

In CAD patients in general, an ischemia-driven revascularization approach guided by PET could result in a reduction in angina severity and a slight improvement in the left ventricular ejection fraction (49). Stulijfzand et al. who examined 69 CTO patients with O-15 water PET, found no correlation between the degree of ischemia and volumetric or functional improvements after CTO PCI but observed a significant ischemic burden reduction (13). In another study, this improvement in myocardial blood flow correlated to the defect size in a baseline PET examination (50). To summarize, PET imaging is a useful tool for evaluating ischemia and viability, with excellent spatial and temporal resolutions.

## Computer Tomography

Computed tomography (CT) angiography has traditionally been used for anatomical evaluations of the coronary tree. With the advent of new-generation scanners with dual-source imaging, 64-row scanners, and modern software techniques, plaque morphology and regional calcium burden evaluations and functional assessments have also become possible (51, 52).

Since CTOs are found in many patients with known or suspected CAD, CT examinations can also contribute to their diagnosis. However, the distinction between a “true” CTO and subtotal stenosis constitutes a diagnostic difficulty. The distal lumen is often contrasted via collaterals, and the spatial resolution of CT is relatively low in comparison to classical angiography. Diagnostic markers such as a lesion length over 9 mm and the so-called reverse attenuation gradient sign have proven to be helpful, enabling a clear diagnosis in most cases (53–55). Apart from pure diagnostics, CT angiography is increasingly used for prognostic assessments and pre-procedural planning of coronary interventions and offers the possibility of follow-up restenosis assessments (56). Blooming artifacts are a limitation caused by severe calcification or stent struts, leading to a potential overestimation of lumen narrowing. This affects diagnosis of CTO as well as follow-up evaluation of the stent patency. Novel techniques and algorithms sought to overcome this limitation (57).

A key task of the pre-interventional examination of CTO patients is the abovementioned viability test. While cardiac CT was previously used for purely anatomical assessments of the coronary tree, it can also be used for hibernation assessments. These are based on classic measures, such as global and regional function, wall thickness, and wall thickening in systole, while assessment methods derived from CMR have not yet found their way into clinical routine. Reduced perfusion can be indicated by reduced contrast during the arterial phase. Similar to the flooding behavior of gadolinium-containing magnetic resonance imaging (MRI) contrast agents, delayed (late) enhancement with iodine-containing contrast media indicates myocardial scarring. A mismatch between underperfusion (arterial phase) and late enhancement indicates a potential intervention target (58). An additional late CT examination (5–15 min after the administration of the contrast medium) is associated with considerable radiation exposure. Currently, only data from experimental and animal studies showing good agreement with CMR scans are available (23, 52, 59, 60).

For the prediction of interventional recanalization success, the Computed Tomography Registry of Chronic Total Occlusion Revascularization (CT-RECTOR) score was established by analogy with angiography-based scores, such as the Japanese Multicenter CTO Registry (J-CTO) and Prospective Global Registry for the Study of Chronic Total Occlusion Intervention (PROGRESS-CTO) scores (61). It is calculated based on the presence of multiple occlusions, a blunt stump, severe calcification in the cross-sectional area of the occluded vessel, tortuosity, anamnestic information from a second attempt, and the presumed duration of the CTO. However, the J-CTO score has also shown applicability to CT angiographies (62).

The actual procedure can also be planned using CT angiography. Depending on factors such as good collateralization or the presence of an intensively calcified proximal cap, the access route (antegrade or retrograde) can be determined, or in the case of severe calcifications over short distances, an early switch to a stiffer wire can be decided. The anticipation of a need for rotablation or debulking devices is a potentially time-saving and patient-friendly factor (63). In a single-center study involving one interventionalist and 73 patients with CTOs, the use of pre-procedural CT angiography for planning significantly increased the recanalization rate in the matched analysis from 64 to 88% (64). In another study with 15 patients with pre-procedural CT planning and 59 patients in a purely classical angiographically controlled group, a stiff wire was chosen significantly more often as the initial wire, and there was less contrast exposure (65). An option after a CT scan that should not be neglected is referral to another center if there is no experience in the use of retrograde maneuvers or foreseeably necessary material is not available.

CT angiography can also be used to detect in-stent restenosis in follow-up investigations. However, as it has shown a high negative predictive value but a low positive predictive value (66–68), its clinical benefit in the case of frequent inconclusive findings, and thus the necessity of an invasive examination with double exposure to radiation and contrast media, must be further investigated in clinical trials. A combination with CT perfusion imaging or fractional flow reserve CT could further increase its value (69, 70).

In summary, pre-interventional multi-slice CT is a useful additional pre-interventional diagnostic tool. If necessary, it should be combined with other examination modalities to detect hibernation. Among all diagnostic modalities, CT is the closest to integrating diagnosis, risk calculation, intervention planning, and follow-up care in one modality.

## Cardiovascular Magnetic Resonance

Cardiovascular magnetic resonance (CMR) is the gold standard for the evaluation of the left and right ventricular volumes and function, as well as ischemia and viability. A major advantage of CMR over nuclear and CT imaging is the lack of a need for ionizing radiation and the better tolerance of gadolinium-containing contrast media compared to iodine-containing contrast media. Its spatial and temporal resolutions are good thanks to modern sequences, such as balanced steady-state free precision imaging, ECG-gating, and breath holding techniques (71). Image quality is getting worse only in patients with severe arrhythmia or non-compliant patients. Real-time imaging without the need for breath holding is evolving to overcome these disadvantages (72).

Ischemia can be visualized using adenosine perfusion CMR. Less contrasted myocardial areas under vasodilator stress indicate a myocardium at risk (73). In CAD patients in general, adenosine perfusion imaging has shown potential for diagnosis and risk prediction (74, 75). Dobutamine stress can be used as an alternative to vasodilatory stress for risk assessment with comparable results (76, 77). However, the role of stress imaging in evaluating CTOs is controversial, as a study found a perfusion deficit in every CTO patient (78). Nevertheless, it can be argued that it may have its place in myocardium risk assessment. Perhaps the ongoing CARISMA_CTO study will provide more clarity on the matter (79).

Two modalities have been developed for viability assessment: low-dose dobutamine CMR (LDD-CMR) and LGE. LDD-CMR is the older method, but it has shown excellent results in predicting functional recovery after revascularization, even in direct comparison with other imaging techniques (80, 81). In low doses, dobutamine increases the regional function of an ischemic or hibernating myocardium (“contractile reserve”). Therefore, an increase in systolic wall thickness with low dobutamine doses is a predictor of functional recovery after revascularization in CAD patients in general. Conversely, in higher doses, the systolic function of an ischemic myocardium deteriorates. A combination with strain imaging is a promising method for improving diagnostic accuracy (82). To date, no studies have evaluated the utility of LDD-CMR prior to CTO revascularization.

LGE imaging is a valuable tool for distinguishing a hibernating myocardium from post-infarction scarring. Five to 15 min after the administration of a gadolinium-based contrast agent, focal deposits of gadolinium in the myocardium show a widening of the extracellular volume, indicating fibrosis and scarring, because of a delayed washout. LGE has shown a good correlation with PET examinations and excellent inter- and intra-observer agreement (83). In stable CAD, the combination of adenosine perfusion imaging and LGE has been shown to improve risk stratification and to help identify patients who will benefit from revascularization (74, 75, 84).

**Figure 3:**
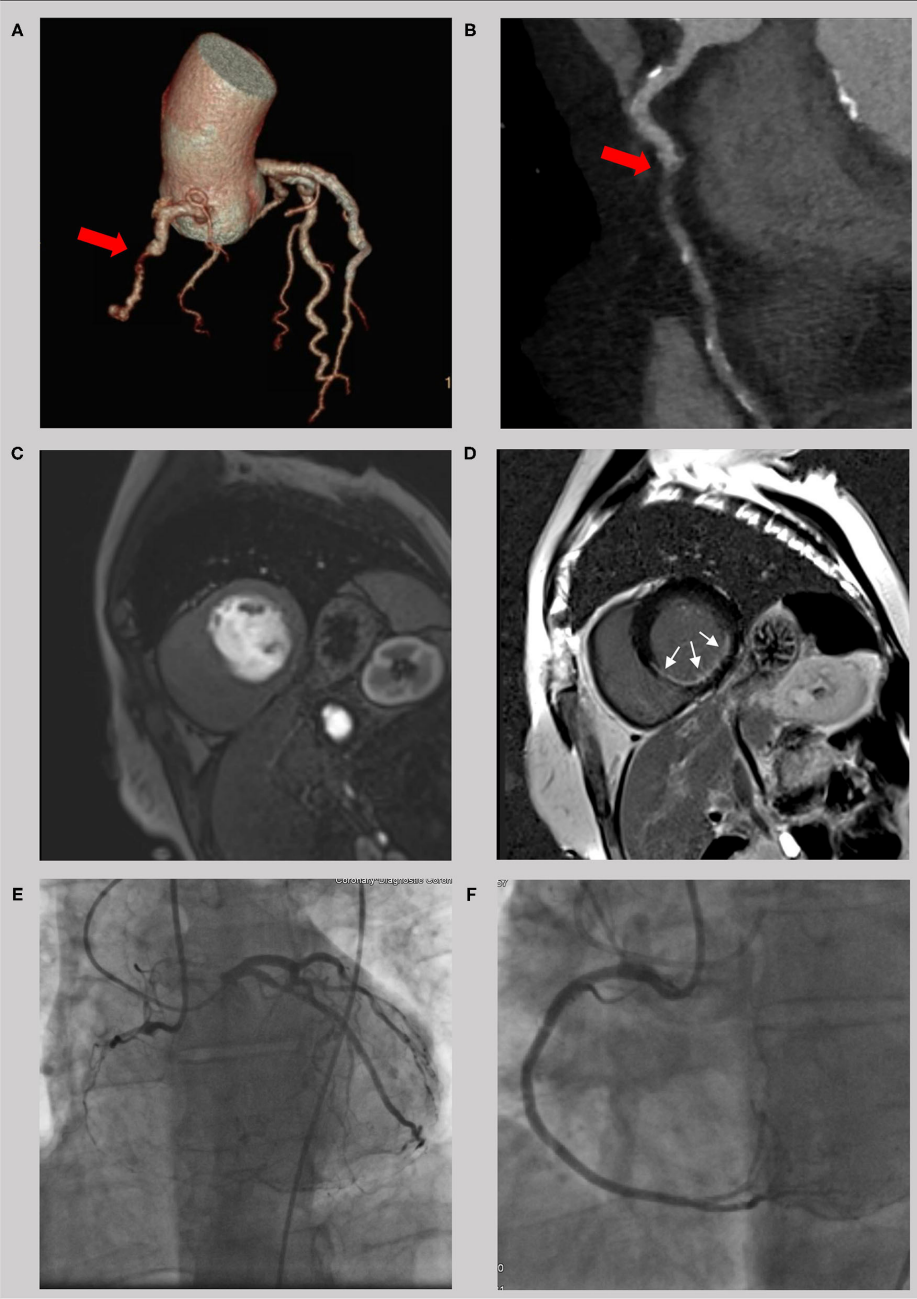
Example of a 56 year old male with a chronic total occlusion of the right coronary artery. The occlusion is seen in the computer tomography coronary angiogram (**A** and **B**, red cross) with a short occlusion length and a small amount of calcification. Because of an impaired regional function with thinning of the left ventricular wall, imaging of ischemia (**C**, first-pass perfusion) and scarring (**D**, late gadolinium enhancement) was done using cardiac magnetic resonance. With a LGE of <50% transmurality (white crosses) and a high symptom burden, interventional revascularization was successfully performed **(E,F)** with significant clinical improvement.

In a trial involving 59 patients with successful CTO PCIs, a pre-interventional LGE extent of <50% was associated with functional recovery of the related segment (85). In another study, CTO PCI was associated with an improvement of myocardial blood flow assessed using CMR and a reduction in the ischemic symptom burden (86). In that study, patients with a transmural LGE extent of more than 75% in most segments in the CTO territory were excluded. Although this approach seems obvious, it should not be seen as absolute, as the perfusion areas of the individual coronaries show significant variations between patients (87). Nevertheless, the two aforementioned studies suggest that a CMR-guided approach to CTO revascularization significantly improves regional blood flow and systolic function and sufficiently reduces patient symptoms. Accordingly, CMR imaging offers great benefits in the pre-interventional diagnostics of CTO patients, with a high prognostic value. An example of the combined use of CT and CMR in a patient with CTO is seen in Figure 3.

For even better workups, new techniques, such as parametric mapping, are currently under investigation. In a first study, the extracellular volume was found to be superior to LGE for functional recovery assessments (88).

Using CMR, patients have already been examined after complex PCI in order to investigate the extent and clinical impact of a peri-interventional infarction. This revealed a relevant proportion of patients with new LGE after PCI, which in turn had a negative impact on the clinical outcome (89). Techniques that are particularly prone to infarction, such as rotablation, could therefore have a negative impact on endpoints in CTO studies (90). Here CMR offers the potential for further investigations.

## Outlook

A central point of future developments, besides a further increase in diagnostic accuracy, will be a reduction in patient exposure to contrast media or radiation. Since CMR typically works without radiation, a further improvement of CMR coronary angiography would be an obvious direction in the quest to overcome the disadvantages of CT in CTO intervention planning. CMR coronary angiography is already feasible for the evaluation of plaque morphology and composition (91–93). At the same time, radiation exposure in SPECT, PET, and CT imaging is now only a fraction of the historically required doses thanks to the constant development of new detectors, collimators, techniques, and software.

Another goal for further improvement of interventional outcomes is the integration of imaging modalities into the actual intervention. Opolski et al. demonstrated the feasibility and safety of an augmented-reality glass that provides the interventional cardiologist with additional information from coronary CT angiography (65). This is yet another indication that CT is the closest to integrating diagnostics, prognostic assessment, planning, and follow-up care.

As already discussed, each modality has its own strengths and weaknesses. Therefore, fusion imaging, such as PET/CT or PET/MRI, could improve the pre-interventional workup of CTO patients. For example, a combination of morphological assessments using CT with metabolic assessments using PET could improve patient selection (94). In a first human study using PET/MRI fusion scans prior to CTO PCI in 49 patients, the combined images predicted functional improvement more accurately than PET or CMR alone (95). However, their high costs and limited access in daily care are clear limitations of these techniques.

In summary, the use of non-invasive imaging for ischemia and viability assessments before interventional recanalization of a CTO is desirable. Although some imaging techniques have clear advantages over others, the selection depends mainly on regional availability and expertise. Better patient selection and prediction of interventional success should be the target of future prospective studies.

## Author Contributions

JK and DB: concept and writing of the manuscript. NE and VP: writing of the SPECT and PET section, proofreading, and image examples. MK, SM, JM, NN, TS, MT, and TT: interpretation of the sources, proofreading, and image example. WR: supervision, concept, and proofreading. All authors contributed to the article and approved the submitted version.

## Conflict of Interest

The authors declare that the research was conducted in the absence of any commercial or financial relationships that could be construed as a potential conflict of interest.

## Publisher's Note

All claims expressed in this article are solely those of the authors and do not necessarily represent those of their affiliated organizations, or those of the publisher, the editors and the reviewers. Any product that may be evaluated in this article, or claim that may be made by its manufacturer, is not guaranteed or endorsed by the publisher.
